# Oleate of Mercury in Obstinate Hepatic Congestions

**Published:** 1885-10-20

**Authors:** C. A. Bryce

**Affiliations:** Richmond, Va.


### OLEATE OF MERCURY IN OBSTINATE HEPATIC CONGESTIONS.

By C. A. Bryce, M. D., Richmond, Va.

Fortunately, slight congestions and derangements of the liver are easily corrected by the early administration of mercurials or vegetable or mineral cholagogues ; therefore, these cases give us but little concern. Occasionally, however, we meet with those inveterate cases in which the list of remedies that we have enumerated fail us utterly, and then we cast about for something to help us out of our dilemma. Quite recently, we had a number of cases of this character, and we desire to place on record the agent which, in our mind, rendered us prompt and efficient service :

### Case I.

W. S——; æt. forty-two ; consulted us "because he could not eat, sleep, sit still, walk about or lie down ; he had headache ; a terrible taste in his mouth, and, in fact, was thoroughly miserable, and had rather be dead than alive." Upon examination, the man's liver was found considerably enlarged, extending down below the false ribs, and toward the median line nearly to the umbilicus. He was given 3 grains of calomel and $\frac{1}{4}$th grain of nux vomica, until four doses were taken. No benefit. Then taraxacum and cream tartar. Ten days trial of this did no good ; then we followed with mineral acid treatment without appreciable benefit.

Before we commenced treating this man, he had been growing worse for three months, and after three or four weeks treatment he had certainly run down a great deal more.

Just at this juncture, it occurred to us to use the oelate of mercury over the region of the liver. We ordered an ointment of a 20-per cent. stength, and directed him to rub it well into the skin every night, and cautioned him to discontinue its use as soon as it produced local irritation. In about a week the patient visited us at our office. His countenance was cheerful, skin clear and healthy-looking, and his step elastic and quick. He said : "That stuff did not blister me as you expected it would, but it just soaked right in and attended to business !" The man was entirely well, and has remained so.

### Case II.

H. B——; about thirty-eight ; white. This man had suffered for six months with general indigestion, anorexia, constant pain over the region of the liver and bowels ; constant diarrhœa and intestinal hemorrhage. The patient had been treated for several months, and, at the time he consulted me, he presented a truly pitiable appearance. His liver was very much enlarged and afforded a heavy, doughy sensation to the fingers of the examiner.

On August 6, I placed him upon the following:

℞. Hyd. chlo. mit.......................................grs. iij,
Ext. taraxaci.........................................grs. xij,
Ext. nux vomica.....................................grs. iij.

M. Ft. pil. no. xii.
S: One every two hours until four are taken.

℞. Hyd. oleat..............................................℥ ss.

S: Apply locally every night.

It is useless to go through details. Within one week the patient returned to our office; the application had had no appreciable effect upon the skin, but all of his symptoms had almost entirely disappeared. After the second day of treatment, his appetite began to return; the diarrhœa and intestinal hemorrhage was entirely relieved, and now (three weeks from commencement of treatment) the patient is entirely well and pursuing his usual avocation.

We attribute the speedy relief in these two cases, and in one subsequently treated, to this special form of medical inunction. It appears peculiar, to us, that in none of these cases did the oleate produce any papular or other eruption upon the skin. It was unquestionably absorbed speedily, and thus affected our patients, when we failed to have desired results from the internal administration of drugs.—*Southern Clinic.*

